# Mathematical modelling of vancomycin-resistant enterococci transmission during passive surveillance and active surveillance with contact isolation highlights the need to identify and address the source of acquisition

**DOI:** 10.1186/s12879-018-3388-y

**Published:** 2018-10-11

**Authors:** Agnes Loo Yee Cheah, Allen C. Cheng, Denis Spelman, Roger L. Nation, David C. M. Kong, Emma S. McBryde

**Affiliations:** 1 Centre for Medicine Use and Safety, Monash University, Parkville, VIC Australia; 2 Department of Infectious Diseases, Alfred Health, Prahran, VIC Australia; 3 Infection Prevention and Healthcare Epidemiology Unit, Alfred Health, Prahran, VIC Australia; 4 Department of Epidemiology and Preventive Medicine, School of Public Health and Preventive Medicine, Monash University, Melbourne, VIC Australia; 5 Microbiology Unit, Alfred Health, Prahran, VIC Australia; 6 Department of Infectious Diseases, Monash University, Melbourne, VIC Australia; 7 Drug Delivery, Disposition and Dynamics, Monash Institute of Pharmaceutical Sciences, Monash University, Parkville, VIC Australia; 8 Pharmacy Department, Ballarat Health Services, Ballarat Central, VIC Australia; 9 Victorian Infectious Diseases Service, Royal Melbourne Hospital, Melbourne, VIC Australia; 10 Department of Medicine, University of Melbourne, Melbourne, VIC Australia

**Keywords:** Active surveillance, Non-rinse chlorhexidine skin cleansing, Prevention, Vancomycin-resistant enterococci, Mathematical modelling

## Abstract

**Background:**

Clinical studies and mathematical simulation suggest that active surveillance with contact isolation is associated with reduced vancomycin-resistant enterococci (VRE) prevalence compared to passive surveillance. Models using pre- and post-intervention data that account for the imperfect observation and serial dependence of VRE transmission events can better estimate the effectiveness of active surveillance and subsequent contact isolation; however, such analyses have not been performed.

**Methods:**

A mathematical model was fitted to surveillance data collected pre- and post-implementation of active surveillance with contact isolation in the haematology-oncology ward. We developed a Hidden Markov Model to describe undetected and observed VRE colonisation/infection status based on the detection activities in the ward. Bayesian inference was used to estimate transmission rates. The effectiveness of active surveillance was assumed to be via increased detection and subsequent contact isolation of VRE positive patients.

**Results:**

We estimated that 31% (95% credible interval: 0.33–85%) of the VRE transmissions were due to cross-transmission between patients. The ratio of transmission rates from patients with contact isolation versus those without contact isolation was 0.33 (95% credible interval: 0.050–1.22).

**Conclusions:**

The majority of the VRE acquisitions in the haematology-oncology ward was estimated to be due to background rates of VRE, rather than within ward patient to patient acquisition. The credible interval for cross-transmission was wide which results in a large degree of uncertainty in the estimates. Factors that could account for background VRE acquisition include endogenous acquisition from antibiotic selection pressure and VRE in the environment. Contact isolation was not significantly associated with reduced VRE transmission in settings where the majority of VRE acquisition was due to background acquisition, emphasising the need to identify and address the source of acquisition. As the credible interval for the ratio of VRE transmission in contact isolated versus non-contact isolated patients crossed 1, there is a probability that the transmission rate in contact isolation was not lower. Our finding highlights the need to optimise infection control measures other than active surveillance for VRE and subsequent contact isolation to reduce VRE transmission. Such measures could include antimicrobial stewardship, environmental cleaning, and hand hygiene.

**Electronic supplementary material:**

The online version of this article (10.1186/s12879-018-3388-y) contains supplementary material, which is available to authorized users.

## Background

The isolation of vancomycin-resistant enterococci (VRE) in patients has become increasingly common [1]. Patients admitted to the haematology-oncology ward are at high risk of VRE colonisation and infection [2], and VRE colonised patients are at greater risk of VRE infections [3]. These infections, such as VRE bacteraemia, are associated with increased mortality and hospitalisation cost [4, 5]. Accordingly, emphasis has been placed on preventing VRE transmission between patients via infection control programs [6].

Active surveillance and subsequent contact isolation has been associated with reduction in VRE transmission in some settings [7–10]. These clinical studies based on observed data had assumed statistical independence in their analysis. Importantly, this assumption may not be true because the probability of a susceptible individual becoming VRE colonised or infected is dependent on the number of VRE colonised or infected patients at a prior time [11]. Whilst published mathematical simulation models describing VRE transmission have provided an understanding of factors that influence VRE transmission in the hospital and outbreak setting [12–18], these studies have not validated their estimated effectiveness with post-intervention surveillance data. Indeed, existing studies on the impact of active surveillance with subsequent contact isolation [7–10, 12, 14] also did not account for unobserved VRE transmission during the passive surveillance period, an issue that can be addressed with Hidden Markov Models (HMMs) or latent state variable models [19]. While the use of statistical methods that account for undetected transmission and dependencies in serial incidence data have been strongly recommended [20, 21], studies of this kind are rare. An extension of the transmission models currently in the literature [18, 19] is required. Thus, to account for undetected VRE transmission during passive surveillance and statistical dependencies, we employed HMMs and Bayesian inference to estimate the effectiveness of active surveillance with contact isolation in reducing VRE transmission.

## Methods

### Patients and setting

This study was approved by the ethics committees of Monash University and The Alfred. Data were collected for patients admitted to the haematology-oncology ward of The Alfred, a major tertiary teaching hospital in Melbourne, Victoria, Australia. The ward has 32 beds for patients with hematological and solid organ malignancies, including allogeneic and autologous bone marrow transplantation. Part of the data on the impact of chlorhexidine skin cleansing on the incidence of VRE colonisation in haematology-oncology patients has been published [22]. The data from the published study [22] corresponds to the active surveillance with contact isolation and active surveillance with contact isolation and chlorhexidine body cleansing phases of this study. The previous report which only included those who had negative rectal swabs on admission as incident VRE cases, found that chlorhexidine skin cleansing was not significantly associated with reduced rate of VRE colonisation but did not account for unobserved VRE transmission and statistical dependencies in the dataset.

### Description of study phases

The study had 3 phases. Data on passive surveillance only was collected for 5 months (21 October 2009 to 21 March 2010). This was followed by active surveillance with contact isolation for 4 months (22 March to 31 June 2010). Following that, in addition to active surveillance with contact isolation, non-rinse chlorhexidine body cleansing using 2% chlorhexidine-impregnated washcloths was implemented for 4 months (1 July to 28 October 2010).

Passive surveillance was the use of routine clinical cultures (blood, sputum, urine) to identify VRE [6]. When a positive clinical isolate for VRE was detected, all other patients in the same ward were screened for VRE by rectal swab. If a new VRE colonised patient who was not previously in contact isolation was identified, surveillance on all potential contacts continued until no further patients were identified with positive VRE rectal swabs. All patients who tested positive for VRE were isolated in single rooms with dedicated bathrooms. As part of routine patient care, the use of gloves, but not gowns, was strongly recommended for these patients and signs were placed on doors notifying staff of isolation requirements. Gowns were only required if contact with body fluids was anticipated.

Active surveillance involved routine rectal swabs for the presence of VRE on all new admissions to the haematology-oncology ward, weekly during hospital stay, and at discharge. Rectal swabs were taken either by nursing staff or patients following instruction, and plated for testing as described previously [22]. Patients who were detected to be VRE positive were contact isolated, as described for passive surveillance above. During the implementation of chlorhexidine skin cleansing, all haematology-oncology patients were provided each day with a pack containing four chlorhexidine washcloths (Clinell Washcloths®, GAMA Healthcare Ltd., London, England) for self-application, as the only form of bathing or after showering with soap and water and drying. These washcloths were applied as described previously [22].

### Data

**Fig. 1:**
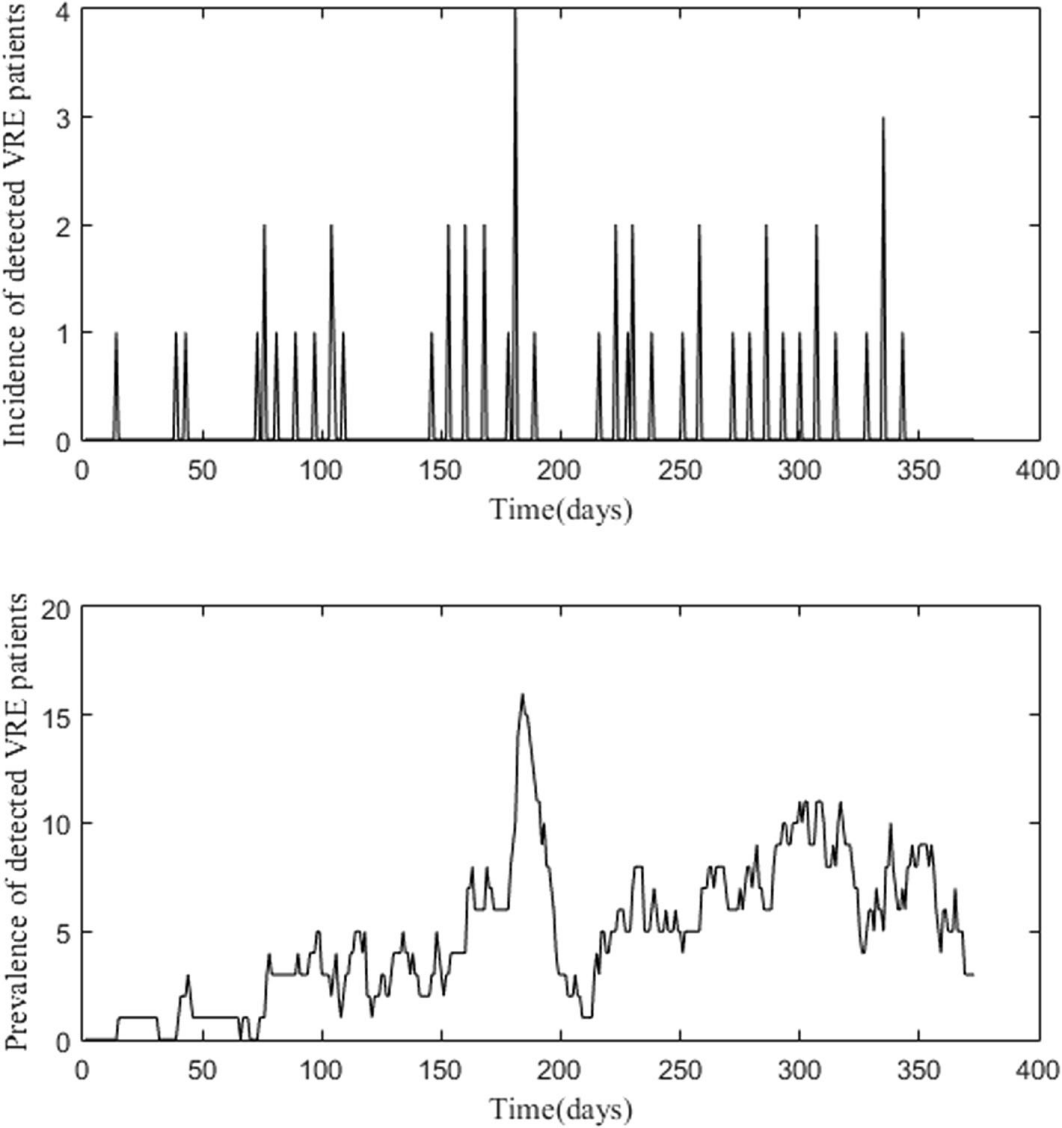
Daily observed (detected) VRE colonisation/infection incidence and prevalence

Microbiology, surveillance and census data for patients admitted to the haematology-oncology ward were obtained retrospectively from the microbiology, infection control and clinical performance databases of the hospital. Incident VRE cases were defined as VRE colonisation/infection in those who were negative on admission and those with unknown VRE status on admission who were detected as VRE positive during their stay in the ward based on passive surveillance or active surveillance (once-weekly VRE rectal swab). Including patients with unknown VRE status on admission ensured a consistent definition of newly-identified incident patients between the active and passive surveillance study phases. This is because in passive surveillance, rectal swabs were not performed on admission, thus patients had unknown VRE status on admission. The prevalence of VRE colonised/infected patient for each day of the study was also utilised in the mathematical model. Patients who were known to be colonised with VRE on admission were defined as those with previous history of VRE within the last 30 weeks [23] and those that were positive within 48 h of admission into the ward. The data used in the present study were the daily observed (detected) VRE incidence and prevalence from 21 October 2009 to 28 October 2010 (Fig. 1). The data are available in the Additional file 1.

### Model structure

**Fig. 2:**
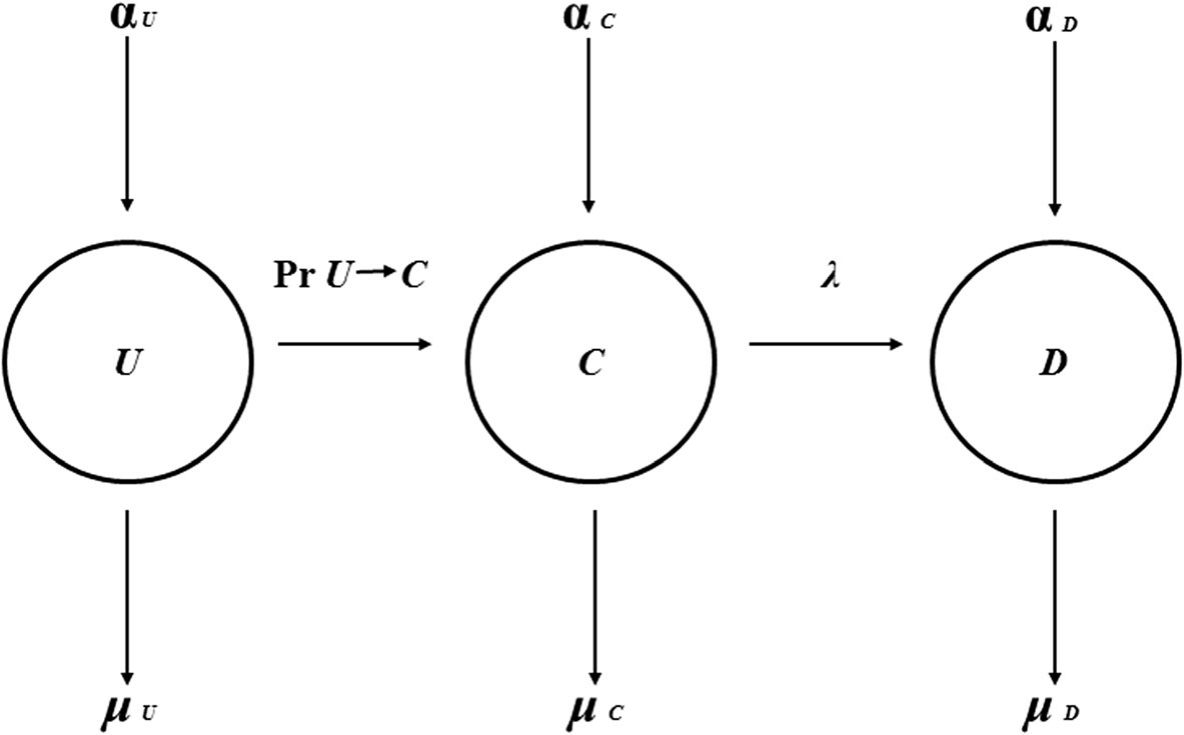
VRE transmission model. *U* = the number of patients who were not known to be colonised/infected (i.e. uncolonised); *C* = the number of patients colonised/infected and not detected (and therefore not contact isolated); *D* = the number of patients who were detected (observed as VRE colonised/infected) and contact isolated; *α*_*U*_, *α*_*C*_ and *α*_*D*_ are the admission rates for *U*, *C* and *D* patients, respectively, and μ_U_, μ_C_ and μ_D_ are the discharge rates for *U*, *C* and *D* patients, respectively. Pr *U → C* = probability of VRE colonisation or infection in uncolonised patients. The detection probability (λ) is expressed as the probability of being detected given that a patient is VRE colonised/infected

A mathematical model (Fig. 2) characterising VRE transmission in the aforementioned strategies was developed to fit the observed surveillance data. This enabled quantification of the effect of active surveillance with or without non-rinse chlorhexidine skin cleansing strategies on VRE transmission. Our model was a modification of the Kermack- McKendrick susceptible-infectious-removed (SIR) model [24, 25] with patient migration and isolation (partial removal) incorporated. The model assumed that the haematology-oncology ward was of fixed size, *N* and had 100% bed occupancy rate. Hence, the number of uncolonised patients was *N-C*-*D*, where *N* was the total number of patients in the ward, and *C*, *D* and *U* are the numbers of hidden (not detected), detected (contact isolated) VRE colonised/infected patients, and uncolonised patients, respectively.

The probability (Pr) of the number of VRE colonised/infected patients (*i*) changing in a small period of time (*h*) is described by the following equations:

1 $$ \Pr\ \left[{C}_{t+h}=i+1\ |\ {C}_t=i\right]=\left[{\beta}_0+{\beta}_1\ C(t)+{\beta}_2\ D\ (t)\right]U(t)\ h, $$

2 $$ \Pr\ \left[{C}_{t+h}=i-1\ |\ {C}_t=i\right]=\left[{\mu}_{\mathrm{c}}\ C(t)\right]\ h, $$

3 $$ \Pr\ \left[{C}_{t+h}=i\ |\ {C}_t=i\right]=1-\Pr\ \left[{C}_{t+h}=i-1\ |\ {C}_t=i\right]-\Pr\ \left[{C}_{t+h}=i+1\ |\ {C}_t=i\right] $$

An uncolonised patient may become VRE colonised/infected by: (i) background acquisition (*β*_*0*_), which may be associated with colonisation/infection from any process that is independent of the number of VRE colonised/infected patients (such as VRE already present on admission and endogenous acquisition from antibiotic selection pressure); (ii) transmission from VRE colonised/infected, but not detected and isolated patients (*β*_*1*_) and; (iii) transmission from VRE colonised/infected, and detected and isolated patients (*β*_*2*_).

The time interval (*h)* was 1 day. To interpret these parameters from a clinical perspective, the mean number of days required for one secondary colonisation per susceptible patient was calculated. The mean number of days to colonisation due to colonised/infected patient cross-transmission for a susceptible patient was 1/[(*β*_*1*_^―^_*C*_) *+ (β*_*2*_^―^_*D*_)], whereby ^―^_*C*_ was the estimated number of hidden (not detected) patients colonised/infected with VRE and ^―^_*D*_ was the observed number of detected patients colonised/infected with VRE. There are separate values of ^―^_*C*_ and ^―^_*D*_ during passive and active surveillance, respectively. The mean number of days for one colonisation from background acquisition (whether from antibiotic selection pressure or cross-transmission from sources other than other colonised/infected patients) for a susceptible patient was 1/ *β*_*0*_.

To estimate *β*_*2*_, we assumed that *β*_*1*_ would change by a ratio of *r* where *β*_*2=*_ *r β*_*1*_*.* The value of *r* was constrained to be any value from 0 to 1.5, denoting that contact isolation reduced transmission if estimated to be between 0 and 1 and increased transmission by no more than 1.5 times if estimated to be between 1 and 1.5_*.*_ The values of *β*_*1*_ and *β*_*2*_ were assumed to be unchanged when moving from passive surveillance to active surveillance with contact isolation. During the period of chlorhexidine skin cleansing, *β*_*1*_ and *β*_*2*_ are replaced by *β*_*3*_ and *β*_*4*_, respectively, to estimate the change in transmission for patients who were cleaned with chlorhexidine washcloths. It was assumed that *β*_*1*_ and *β*_*2*_ would change by a ratio of *v*, whereby *β*_*3*_ = *v β*_*1*_ and *β*_*4*_ = *v β*_*2*_*.* Chlorhexidine skin cleansing was assumed to have no impact on reducing VRE colonisation and infection in haematology-oncology patients and the value of *v* was assumed to be 1 in the base-case model, based on the results of a non-statistically significant reduction in VRE acquisition from a previous study [22]. The value of *v* was varied in the sensitivity analysis. The value of *β*_*0*_ was assumed to not change between passive surveillance, active surveillance and chlorhexidine skin cleansing.

The admission rates for *U*, *C* and *D* patients are *α*_*U*_, *α*_*C*_ and α_D_, respectively and discharge rate for *U*, *C* and *D* patients are μ_U_, μ_C_ and μ_D_, respectively (Fig. 2). Estimates of these admission and discharge rates are not required as we assumed a population of constant size (where the net inflow of uncolonised patients equals the outflow of colonised patients). Hence rather than estimating *α*_*U*_, *α*_*C*_ and α_D_, we only estimated the net change in *U*, *C* and *D* at each time step. The discharge probability of detected VRE colonised/infected patients per day was estimated as the reciprocal of the mean time from first identification of colonisation/infection to discharge (10.4 days). We assumed the length of stay of the undetected patients, 1/*μ*_*C*,_ is the same as for the detected patients, 1/*μ*_*D*_.

Once detected as VRE colonized/infected, patients were assumed to remain colonised for the duration of their hospital stay and to contribute to the risk of VRE transmission. This assumptions is justified, given the observation that duration of VRE carriage is considerably longer than length of hospital stay [26]. The patient population was assumed to be homogenous in terms of susceptibility for colonisation [27], and homogenous mixing of patients was assumed. VRE colonised and infected patients were also assumed to contribute in the same manner to colonisation pressure and thus risk of VRE tranmission in the ward [11]. Contact isolation of VRE-positive patients was assumed to commence on the day of positive VRE culture. VRE acquisition pressure due to antibiotic use was assumed to be identical for all study phases, and was not part of the model.

### Bayesian framework

**Fig. 3:**
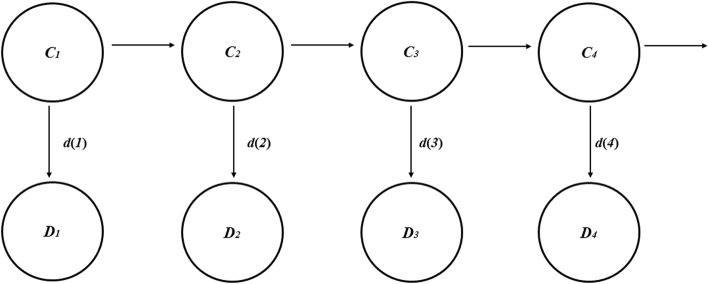
Hidden Markov model. *C* = the estimated number of detected and undetected VRE colonised/infected patients in the underlying hidden states at each time points (*t* = 1,2,3,….); *D* = the number of patients detected as VRE colonised/infected. The transition model linking the hidden states are represented by the horizontal lines, whereas the observation model linking the hidden states and the corresponding observed data are represented by the vertical lines. The probability of observing *d* new infections on day *t*, *d(t)* is assumed to be a Poisson distribution given by *d(t)~* Poisson (*λ*). Each day, the hidden states were updated based on observed new acquisitions, admissions and discharges from the dataset, and also via the transition probabilities in Eqs. (1) to (3)

All analyses were performed in MATLAB (Version 2015b, MathWorks, Natick, MA, USA). A structured HMM framework with transition and observation components was used (Fig. 3). The HMM was used to generate a likelihood of the transmission parameters given the observed data, integrated over the hidden states. This likelihood was then used along with prior probabilities to generate posterior probabilities for the model parameters. Posterior distributions for the daily prevalence and incidence of colonised and infected patients (including cryptic transmission) was also estimated. Hence we were able to infer the underlying number of patients and transmission rates [19, 27].

### Observation model

The observation model describes the probability of VRE detection based on the true (unobserved) prevalence of VRE colonisation/infection for each of the three phases. The Poisson distribution was used to describe the probability relationship between the observations and corresponding hidden states. As detected incident patients were contact isolated, at each observation, the detection parameter (*λ*) describes the daily probability of detecting an incident VRE colonised/infected patient. The model assumed that for a given colonisation status, each patient has the same constant probability of being detected. For passive surveillance only, the daily probability of detection is described by *λ*. During active surveillance, *λ* is replaced by *λ*_*2*_*,* allowing for a different detection probability. We assumed that during active surveillance, the daily probability of a patient detected as a new VRE case would increase. The increase in the daily probability of a patient detected as a new VRE case is denoted *PD*. The value of *PD* was assumed to be based on the sensitivity of the VRE rectal swab. Literature review suggested that the sensitivity of VRE rectal swab was estimated to be between 0.58 [28] and 0.97 [29]. As the rectal swab was only performed once a week during active surveillance, the daily average increase in detection per undetected VRE case was estimated based on the average sensitivity of a VRE rectal swab divided by 7 (i.e. 0.775/7). Thus *PD* was assumed to be 0.775/7. The daily probability of detecting a new VRE case during active surveillance (*λ*_*2*_) is equal to this active case detection probability plus the probability that active case detection does not occur but passive case detection occurs. That is the daily probability of detection per undetected VRE case during active surveillance (*λ*_*2*_) = *PD* + (1- *PD*)(*λ*). The value of *PD* was varied in the sensitivity analysis.

### Transition model

The hidden state transition component of the HMM is given in Fig. 3. Hidden patients include those that are not detected but are colonised/infected with VRE. Estimates of transmission parameters were made using the likelihood as per Baum’s recursion algorithm and Bayesian inference tools described below.

The effectiveness of active surveillance in preventing VRE transmission was assumed to act via increased detection, leading to transition into isolation (*D*). Unknown parameter values were estimated within a Bayesian framework using a Markov Chain Monte Carlo (MCMC) algorithm [30]. In the MCMC estimation, *θ* = {*β*_*0*_, *β*_*1*_, *β*_2_, *λ*} was the vector of the model parameters. The likelihood of the observed data, Y, is given by determining the joint likelihood of the observations and the hidden states, X, Pr (Y|*θ*, X) Pr(X|*θ*), summed over all possible hidden states. This was determined using Baum’s recursion algorithm (Additional file 2) [31]. The posterior probability distributions of the transmission parameters *β*_*0*_, *β*_*1*_, *β*_2,_ and *λ* were estimated using MCMC algorithms (Additional file 3). Markov chain convergence was assessed via visual analysis of trace plots [32] and calculation of the Gelman-Rubin convergence statistic [33].

The expected number of acquisitions due to cross-transmission during a one-day period (between time t_*k*_ and time t _*k + 1*_) is *β*_*1*_ *U*_*k*_ *C*_*k*_ + *β*_*2*_ *U*_*k*_ *D*_*k*_. Each time point corresponds to 1 day and *n* is the total number of study days. The expected number of acquisitions (both cross-transmission and background acquisition) is *β*_*0*_ *U*_*k*_ + *β*_*1*_ *U*_*k*_ *C*_*k*_ + *β*_*2*_ *U*_*k*_ *D*_*k*_. Hence the proportion of VRE colonisation/infection acquired via cross-transmission between patients, *p*, was

$$ p=\frac{\sum \limits_{k=1}^n{\beta}_1{U}_k{C}_k+{\beta}_2{U}_k{D}_k}{\sum \limits_{k=1}^n{\beta}_0{U}_k+{\beta}_1{U}_k{C}_k+{\beta}_2{U}_k{D}_k} $$

The effectiveness of active surveillance and subsequent contact isolation, *r*, was assessed by estimating the ratio of transmission rates in patients with contact isolation versus those without contact isolation, calculated as *β*_*2*_/*β*_*1*_.

### Model selection

As shown in Table 1, we evaluated models with background acquisition rate (*β*_0_) (Models 2, 4 and 5) and models without *β*_0_ (Models 1 and 3). Models with *β*_0_ suggest that VRE can be acquired sporadically as well as through patient-to-patient transmission, whereas models without *β*_0_ suggest that VRE can only be acquired through patient-to-patient transmission. A number of models were parameterised using combinations of assumed and estimated parameters (Table 1). To help with model selection, we considered whether the model converged, and the Bayesian information criterion (BIC), the Akaike information criteria (AIC) and Deviance information criterion (DIC) were calculated. The BIC has been shown to be suitable for model selection of Bayesian HMMs [34]. It is based on the trade-off between the model’s goodness-of-fit and the corresponding complexity of the model [34]. Preference is for the model with the lowest BIC value [34]. As the preferred diagnostic for model selection of HMMs remains unresolved [34], the AIC and DIC previously shown [18, 35] to be appropriate in this setting were also calculated. Similar to the BIC, the model with the lower values of AIC and DIC are preferred [36].

**Table 1:** Comparison of different models

Model number	*β*_0_	*β*_1_	*β*_2_	*v*	*λ*	*λ*_2_	Convergence achieved
1	Assume *β*_0_ = 0	Estimated	Estimated	Estimated	Estimated	Estimated	No
2	Estimated	Estimated	Estimated	Estimated	Estimated	Estimated	No
3	Assume *β*_0_ = 0	Estimated	Estimated	Assume *v* = 1	Estimated	Assume *λ* and *λ*_2_ related by *PD*, whereby *PD* is 0.775/7	No
4	Estimated	Estimated	Estimated	Estimated	Estimated	Assume *λ* and *λ*_2_ related by *PD*, whereby *PD* is 0.775/7	No
5	Estimated	Estimated	Estimated	Assume *v* = 1	Estimated	Assume *λ* and *λ*_2_ related by *PD*, whereby *PD* is 0.775/7	Yes

## Results

Convergence was not observed in Models 1, 2, 3 and 4. When assumptions were made on the parameters *v, λ* and *λ*_2_ (Model 5), model fit improved and convergence was achieved. Thus Model 5 (where acqusition may be by cross transmission or via other sources) best fits our study data. Although we set out to calculate the BIC, AIC and DIC values of the different models, these values were not calculated or presented as only Model 5 converged.

We estimated that 6% (2/32) and 3% (1/32) of hidden (not detected) patients were colonised/infected (^―^_*C*_) with VRE at any time point during passive surveillance and active surveillance with contact isolation, respectively. Based on the dataset, 6% (2/32) and 22% (7/32) of patients were detected to be colonised/infected with VRE (^―^_*D*_) at any given time point during passive surveillance and active surveillance with contact isolation, respectively.

**Fig. 4:**
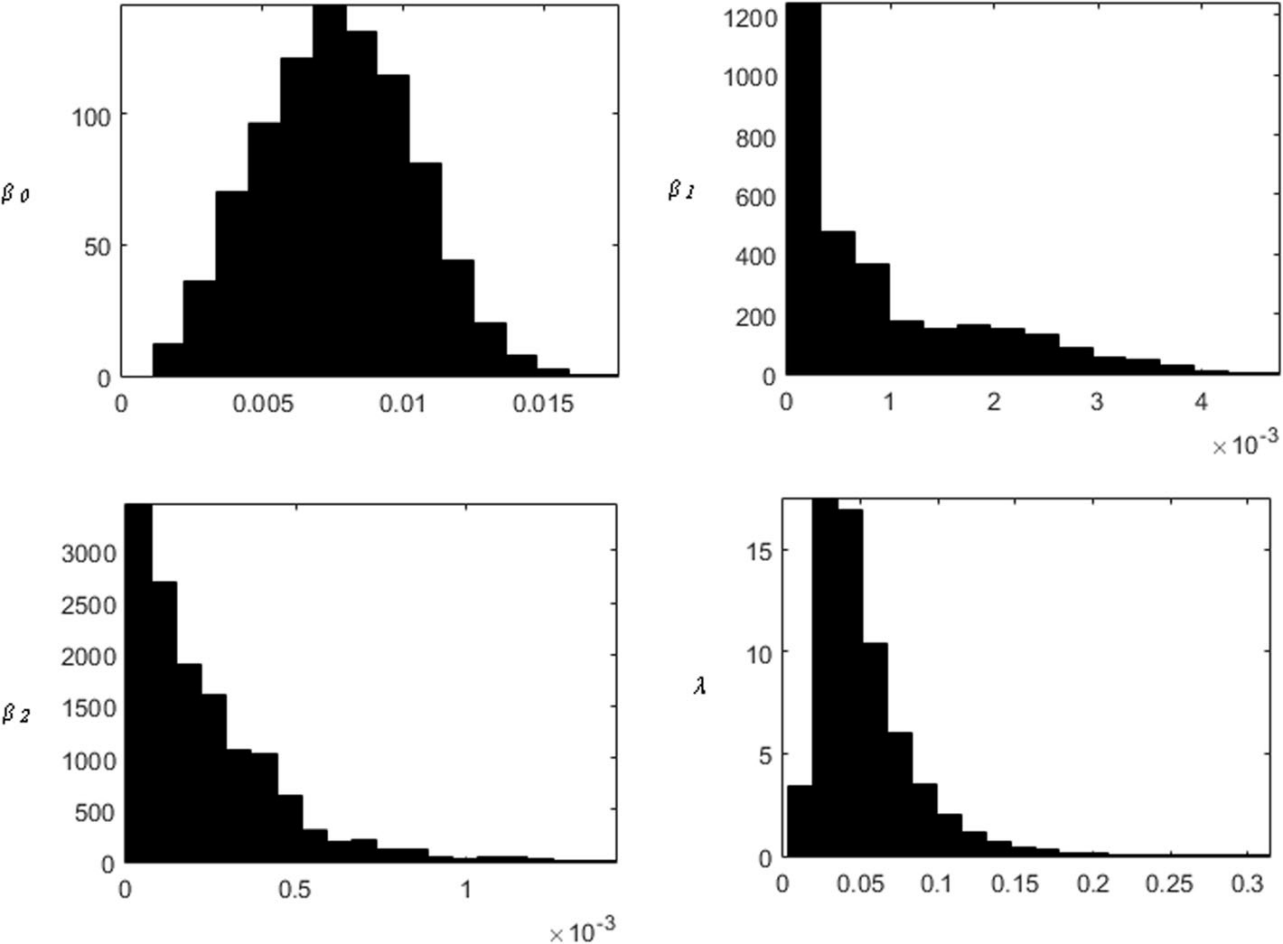
Posterior probability density of parameter estimates

Estimates of the transmission parameters *β*_*0*_, *β*_*1*_, *β*_2_, and *λ* for Model 5 are in Table 2. Posterior probability distributions of the estimated parameters are in Fig. 4. We estimated that the background acquisition rate (*β*_*0*_) was 0.0076 (95% credible interval 0.0026 to 0.013). That is, on average, a susceptible patient will become colonised as a result of background acquisition every 131 patient days (95% credible interval: 78 to 379 days). The estimated cross-transmission coefficient without (*β*_*1*_) and with contact isolation (*β*_*2*_) was 0.00049 (95% credible interval 4.9 × 10^− 6^ to 0.0033) and 0.00017 (95% credible interval 1.2 × 10^− 6^ to 0.00082), respectively. That is, based on the numbers of hidden and detected colonised/infected patients in the study, on average, a susceptible patient became colonised as a result of cross-transmission every 758 days (95% credible interval: 121 to 82,234 days) and 622 days (95% credible interval: 117 to 78,312 days) in passive surveillance and active surveillance with contact isolation, respectively. The ratio of transmission rates in patients with contact isolation versus those without contact isolation (*r)* was 0.33 (95% credible interval: 0.050 to 1.22). Thus, we estimated lower rates of transmission from patients who were contact isolated, but there remains significant possibility that the transmission rates in contact isolation are not lower. The estimated detection probability during passive surveillance (*λ)* was 0.044 (0.016 to 0.14). The proportion of VRE colonisation/infection that was acquired via cross-transmission between patients, *p*, was estimated to be 0.31 (95% credible interval: 0.0033 to 0.85) suggesting that the majority of the VRE acquisition in the haematology-oncology ward was estimated to not be associated with patient-to-patient transmission.

**Table 2:** Estimated model parameters for Model 5

Parameters	Symbol (unit)	Median values (95% credible intervals)
Background acquisition coefficient	*β*_0_ (×10^− 4^) (/susceptible/day)	76 (26 to 130)
Cross-transmission coefficient in patients who were not contact isolated	*β*_1_ (×10^− 4^) (/VRE colonised or infected patient that was not contact isolated/susceptible/day)	4.9 (0.049 to 33)
Cross-transmission coefficient in patients who were contact isolated	*β*_2_ (×10^−4^) (/VRE colonised or infected patient that was contact isolated/susceptible/day)	1.7 (0.012 to 82)
Probability of detection for passive surveillance	λ	0.044 (0.016 to 0.14)
Ratio of VRE transmission with contact isolation versus without contact isolation	–	0.33 (0.050 to 1.22)
Proportion of VRE colonisation/infection that was acquired via cross-transmission	(%)	0.17 (0.0015–0.72)

## Discussion

In this study, we have accounted for unobserved VRE transmission during passive surveillance and statistical dependencies in VRE transmission data. We estimated that the majority of the VRE acquisition in this haematology-oncology ward was due to background VRE acquisition, whereas the proportion of VRE colonisation/infection that was acquired via cross-transmission between patients was 31% (95% credible interval: 0.33% to 85%). In the haematology-oncology (study) ward, we estimate that factors independent of the number of VRE patients account for 69% of VRE acquisitions. The types of events that lead to acquisition that is independent of cross-transmission include endogenous acquisition from antibiotic selection pressure and VRE in the environment. A major strength of this study is that, unlike earlier studies using non-Bayesian methods [7, 8, 10], we inferred the number of undetected VRE colonisation cases using a HMM framework and Bayesian analysis. Our approach also accounted for data dependencies; the fact that VRE transmission at a certain time is dependent on number of VRE colonised and/or infected patients at an earlier time.

The aim of this study was to assess the pre- and post-intervention surveillance data to quantify the impact of contact isolation on VRE transmission in the ward. However due to the low VRE transmission observed throughout the study period, we were unable to assess the effectiveness of contact isolation. Our model estimated that VRE transmission was reduced in patients who were contact isolated compared with those in the open ward (relative infectiousness = 0.33), but the credible interval for this parameter was wide and crossed unity. Hence while the effect size is potentially large, we cannot conclude definitively that active surveillance and contact isolation reduced transmission. Additional data including more time points and differing levels of incidence of VRE would allow greater precision of this estimate.

Even if the effect size is as large as the point estimate of 0.33, the proportion of acquisitions through this route is estimated to be only 31%, hence the overall impact is limited in this setting. Thus infection control measures such as antimicrobial stewardship, and environmental cleaning may be more important than VRE detection and contact isolation in settings where the majority of VRE transmission was estimated to be due to background acquisition. This emphasises the need to identify and address the source of VRE acquisition when implementing measures to prevent VRE acquisition.

Our finding was consistent with a study in another hospital in Melbourne which found that in a significant proportion of patients, the *vanB* transposon was generated from multiple different events within a patient rather than cross-transmission from a clonal outbreak [37]. We may have also observed our results because of one or more of the following factors: our study was conducted in the non-VRE outbreak setting, we included both VRE colonised and infected patients in the study rather than infected patients alone and other infection control measures were not optimised. Previous studies that found active surveillance with contact isolation to be more effective than passive surveillance were conducted in different settings [8, 10], assessed different study outcomes [7, 9], .and active surveillance with contact isolation was implemented as part of a multiple strategy intervention [10].

Several assumptions were made in our model. In the observation model, the Poisson distribution was used to describe the probability relationship between the observations and corresponding hidden states. The Poisson distribution was chosen as we utilised incidence data [18]. Limited information is available on the increase in probability of VRE detection during active surveillance (*PD*) and the effectiveness of non-rinse chlorhexidine skin cleansing (*v*) on VRE transmission in the haematology-oncology setting. We allowed for the uncertainties in these variables by randomly drawing the value of *PD* from a beta distribution based on the adjusted values of 0.083 and 0.14 (based on sensitivity of VRE rectal swab between 0.58 [28] and 0.97) [29], and *v* from a beta distribution of between 0.06 and 1.59 [38]. As the credible intervals were wide for *β*_*1*_ and *β*_*2*_, the degree of uncertainty was large. Thus the estimation of *β*_*1*_ and *β*_*2*_ may not be fully interpretable given the large degree of uncertainty in these parameters. Although contact isolation has not always been associated with reductions in VRE transmission [39, 40], we did not feel that it was plausible that contact isolation would increase VRE transmission by more than 50%. Therefore we constrained the ratio of transmission in patients with contact isolation versus those without contact isolation (*r* = *β*_*2*_/*β*_*1*_) to be between 0 and 1.5. A constant discharge rate was assumed throughout the study implying an exponential distribution of LOS. As our data showed that the length of stay distribution was right-skewed with a long tail and had a mode that was close to zero, the assumption of a constant discharge rate was plausible. Given that patients were found to remain colonised with VRE up to a few years after first detection [26, 41], we assumed that VRE colonised/infected patients remain so until discharge.

The current model enabled estimation of VRE transmission during our study phases by using actual data on VRE colonisation and infection; however, we were not able to consider VRE strain sequencing. The relative contributions of antibiotic use, compliance with contact isolation, co-morbidities, and illness severity to the risk of VRE transmission were not measured and were assumed to be the same for all the study phases. Such factors could be incorporated into future mathematical models. Due to the lack of data, we assumed that all uncolonised patients had the same susceptibility to VRE colonisation/infection. Future models could also incorporate heterogeneity in susceptibility of patients to VRE colonisation/infection based on certain risk factors in their analysis. To simplify the model, homogenous mixing of patients within the study wards was assumed. Whilst the impact of non-homogenous patient mixing on results can be explored in future studies, the increase in model complexity and reduced precision associated with such studies may not be favourable.

## Conclusions

This study found that patients who were contact isolated did not have a significant reduction in VRE transmission rate compared to those who were not contact isolated and that most acquisition was not from cross transmission of VRE in our setting. These findings highlight the importance of identifying and addressing the source of VRE acquisition in the implementation of measures to prevent VRE acquisition. When the majority of VRE acquisition is due to background acquisition, infection control measures other than active surveillance with contact isolation (such as antimicrobial stewardship, environmental cleaning and hand hygiene) need to be optimised.

## Additional files

Additional file 1: Raw incidence and prevalence data. (XLSX 15 kb)

Additional file 2: Likelihood computation. (DOCX 36 kb)

Additional file 3: Monte Carlo Markov Chain algorithm. (DOCX 19 kb)

## Abbreviations

AIC: Aikaike information criterion
BIC: Bayesian Information Criterion
DIC: Deviance Information Criterion
HMMs: Hidden Markov Models
PD: Probability detected
Pr: Probability
SIR: Susceptible-infectious-removed
VRE: Vancomycin-resistant enterococci
