# Supplementary material for: Mathematical modelling of vancomycin-resistant enterococci transmission during passive surveillance and active surveillance with contact isolation highlights the need to identify and address the source of acquisition
Source: BMC Infect Dis. 2018 Oct 11;18:511. doi: 10.1186/s12879-018-3388-y (PMC6182842; doi:10.1186/s12879-018-3388-y)
Supplement: Supplementary file 2 — Likelihood computation. (DOCX 36 kb) [file 12879_2018_3388_MOESM2_ESM.docx]

**Additional file 2**

**Likelihood computation**

The probability of the full dataset of *n* observations (*D_1_*, *D_2_*, …, *D_n_*) and a particular sequence of hidden states, *C_1_*, *C_2_*, … , *C_n_* is given by

Pr(*D_1_*,…, *D_n_*, *C_1_*,…, C_n_) = Pr (*C_1_*) Pr (*D_1_* | *C_1_*) Pr (*C_k_* | *C_k_*_-1_) Pr (*D_k_* | *C_k_*).

The likelihood calculation of this single permutation of hidden states requires *2n* computations even after the matrix exponential of the generating matrix has been evaluated. The full likelihood of the data over all the states is

Pr(*D_1_*,…, *D_n_*| *θ*) = ,…,( *C_1_*) Pr (*D_1_* | *C_1_*) Pr (*C_k_* | *C_k_*_-1_) Pr (*D_k_* | *C_k_*), where *θ* is the vector of model parameters. This requires 2n(N + 1)^n computations for one likelihood evaluation.([1](#_ENREF_1)) This intractable calculation (with n = 372 and N = 33) can be simplified using Baum’s recursion technique,([2](#_ENREF_2)) shown below.

The forward recursion involves simplifying the likelihood computations by

considering a partial observation sequence and estimating the likelihood of ending in each possible state {0, *N*}. Let φ_k_(*i*) be the probability of the partial observation sequence *(D_1_, D_2_, … , D_k_)* produced by all possible state sequences that end in state *i*. The probability is given by

φ_k_(*i*) = L(*D_1_*,…, *D_n_*,… , *D_k_*; *C_k_*= *i* | *θ*);

Let *σ* be the (size *N*+1) vector of probabilities of the first state, *σ_i_* = Pr(*C_1_*= *i*). In the forward recursion method of likelihood computation, the value of *σ* needs to be determined in the absence of data. The stationary distribution of the transition matrix can be used for this.([3](#_ENREF_3))

The likelihood of the first state being *i* and first observation, *Y_1_*, being *y_1_* is given by φ_1_(*i*) = *σ_i_* Pr(*D_1_* | *C_1_*= *i*). The forward recursion formula is then applied. We multiply every state partial likelihood, φ_k_(*i*) by the transition probability $\Gamma$*ij* and by the probability of the *k^th^* data point given the hidden state *j*. This results in a vector of probabilities which is then summed to determine φ_k+1_(*j*). Thus the full likelihood is given by Pr(*D_1_*,…, *D_n_* | *θ*) = [φ_n_(*i*) = (*σ*Pr (*D_1_* | *C_1_*) $\Gamma$ Pr (*D_k_* | *C_k_*)) ]*v*, where *v* is a column vector, with each element equal to unity. See Petrushin,([4](#_ENREF_4)) for a detailed discussion of the forward and backward recursion formulae.

**Some peculiarities of this particular application.**

Most Hidden Markov Models can assume that the observational model is dependent on the hidden state but the reverse is not true. That is observations of the data do not in turn causally affect the model transitions from one state to another. However, in this study, the hidden state was predictably dependent on the observations. Patients were isolated once detection took place. In other words, observations of prevalent detected (*D*) patient modified the hidden colonised (*C*) patient state-space in the direction of – *D* state and modified the state space of isolated patients to + *D*. To account for this, the following modifications were made.

1. After each observation of incident VRE detection (*D_k_*), it was assumed that from the end of that day, the individual was no longer in the *C* state, but in the *D* state. Hence the vector of state partial likelihood, φ_k_(*i*)**,** had to be updated to φ_k_(*i*)^updated^, using the following formula; φ_k_(*i*)^updated^ = φ_k_(*i + D_k_*), for values of *i≤N- D_k_* and φ_k_(*i*)**^*^** = 0, for values of *i>N- D_k_* ***.*** For example, if there is a partial likelihood at observation *k* of 6 prevalent colonised/infected patients being on the ward, φ_k_(6), and at observation point *k,* 2 of these were detected as incident cases and removed into the isolation compartment, the partial likelihood given by φ_k_(6) is transferred to φ_k_(4)^updated^ using the formula φ_k_(4)^updated^ = φ_k_(4+2).
2. There were several different transitional probability matrices (TPMs), one for each value of *D*. In this study, *D* took on values from 0 to 16 during passive surveillance and active surveillance with contact isolation phases (17 TPMs), and *D* took on values from 0 to 12 during active surveillance with contact isolation and chlorhexidine skin cleansing (13 TPMs). On day *k*, the correct TPM_*k* had to be selected based on the number of *D* on day *k*. These numbers for *D* were based on observed numbers of detected patients during the study.

**References**

1. Le Strat Y, Carrat F. Monitoring epidemiologic surveillance data using hidden Markov models. Stat Med. 1999;18:3463-78.

2. Baum LE, Petrie T, Soules G, Weiss N. A maximization technique occurring in the statistical analysis of probabilistic functions of Markov chains. Ann Math Stat. 1970;41:164-71.

3. MacDonald I.L., Zucchini W., Hidden Markov and other models for discrete-valued time series. London: Chapman and Hall, 1997.

4. Petrushin VA (2000) Hidden Markov Models:Fundamentals and Applications, Part 2: Discrete and Continuous Hidden Markov Models. Online Symposium for Electronics Engineer 2000. Available <http://www.eecis.udel.edu/~lliao/cis841s06/hmmtutorialpart2.pdf>. Accessed 17 January 2017.
