# Supplementary material for: Mathematical modelling of vancomycin-resistant enterococci transmission during passive surveillance and active surveillance with contact isolation highlights the need to identify and address the source of acquisition
Source: BMC Infect Dis. 2018 Oct 11;18:511. doi: 10.1186/s12879-018-3388-y (PMC6182842; doi:10.1186/s12879-018-3388-y)
Supplement: Supplementary file 3 — Monte Carlo Markov Chain algorithm. (DOCX 19 kb) [file 12879_2018_3388_MOESM3_ESM.docx]

**Additional file 3**

**Monte-Carlo Markov Chain algorithm**

The Monte-Carlo integration algorithm given below was used to estimate the model parameters. Monte Carlo Markov Chain (MCMC) algorithm has the following steps:

**1.** Assume the prior probability for *β_0_*, *β_1_*, and *β*_2_ to be a uniform distribution from 0 to 0.1, *λ* to be a uniform distribution from 0 to 1. These uniform uninformative priors were used because little prior information was known, except that negative values and values greater than 0.1 and 1, respectively, are implausible.

**2.** Initialise parameter selection, *θ*.

**3.** Assign the prior probability of the hidden states. A discrete uniform distribution of (0,…, *N*) was used.

**4.** Initialise each hidden state with the corresponding observed data and the Poisson observation model.

**5.** Determine the likelihood, Pr(*Y***|** *θ*) using the Baum’s recursion algorithm in Additional file 1.

**6.** Propose a new *θ*′ using a simple random walk, using a step size from the normal distribution N(0, 0.02), N(0, 0.008), N(0, 0.007), and N(0, 0.1) for *β_0_*, *β_1_*, *β*_2_, and *λ*, respectively, after transforming the variables using the logit function.

**7.** Accept *θ*′ using a Metropolis step with the acceptance probability,

P_acc_ = min

**8.** Iterate by returning to step 5 to 7.

**9.** Burn in using 130,000 iterations. Use the following 70,000 updates to estimate the posterior probability distribution for the model parameters.

**10.** Repeat steps 2 to 9 for four other Markov chains, each with different initial values.

**11.** The Gelman-Rubin diagnostic was calculated for all five Markov chains to test for convergence.([1](#_ENREF_1)) The Gelman-Rubin diagnostic value of between 1.0 and 1.2 for all estimated parameters showed that 70,000 updates were sufficient to achieve convergence and obtain precise estimates of the unknown parameters.([1](#_ENREF_1))

**Reference**

1. Gelman A, Rubin DB. Inference from iterative simulation using multiple sequences. Stat Sci 1992;7:457-511.
